# Right cardiac failure and pulmonary hypertension secondary to tonsil hypertrophy in a 3-year-old boy: a case report

**DOI:** 10.11604/pamj.2025.50.2.39678

**Published:** 2025-01-02

**Authors:** Mehdi Berrajaa, Abdeladim Babakhouya, Mohamed El Minaoui

**Affiliations:** 1 Cardiology Department, Souss-Massa University Hospital, Faculty of Medicine of Agadir, University Ibn Zohr, Agadir, Morocco,; 2 Pediatrics Department, Mohammed VI University Hospital, Faculty of Medicine of Oujda, Oujda, Morocco

**Keywords:** Childhood obstructive sleep apnoea syndrome, tonsil hypertrophy, right heart failure, pulmonary hypertension, case report

## Abstract

In children, the prevalence of obstructive sleep apnoea syndrome (OSAS) is 2 to 3%. Pulmonary hypertension-related OSAS in the context of tonsil hypertrophy is a well-documented phenomenon. However, cases combining pulmonary hypertension and right cardiac failure have rarely been reported. The management of such cases is based on adenotonsillectomy with an important subsequent impact on the right ventricle and left ventricle functions. Herein, we report a 3-year-old boy with right cardiac failure and pulmonary hypertension due to OSAS that was secondary to bilateral tonsil hypertrophy. The patient has well evolved with complete resolution of OSAS, and cardiac and respiratory alteration after adenotonsillectomy. Our reported case should be a reminder for otolaryngologists, pediatricians, and cardiologists to recognize this particular condition.

## Introduction

Childhood obstructive sleep apnoea syndrome (OSAS) occurs in 2 to 3% of children [1]. It is a known etiology of pulmonary hypertension [2]. Adenotensillar hypertrophy and palatine tonsillar hypertrophy are the most frequent cause of OSAS in children [3]. Case reports of tonsil hypertrophy being complicated by pulmonary hypertension and right heart failure are rare in the literature. The management of these patients is based on adenotonsillectomy since this latter provides significant benefits, especially in cases of non-response to medical treatment [4].

We report a case of a 3-year-old boy with right cardiac failure and pulmonary hypertension due to OSAS that was secondary to bilateral tonsil hypertrophy. The patient has well evolved with complete resolution of OSAS, and cardiac and respiratory alteration after adenotonsillectomy.

## Patient and observation

**Patient information:** we report a 3-year-old boy who presented rapidly progressive respiratory distress evolving for 2 weeks. The patient was born at fu

ll term, with a birth weight of 3.5 kg. He has no particular medical history. He has no known syndromic disorders or hereditary diseases. The mother reports no apnea-like episodes or snores.

**Figure 1:**
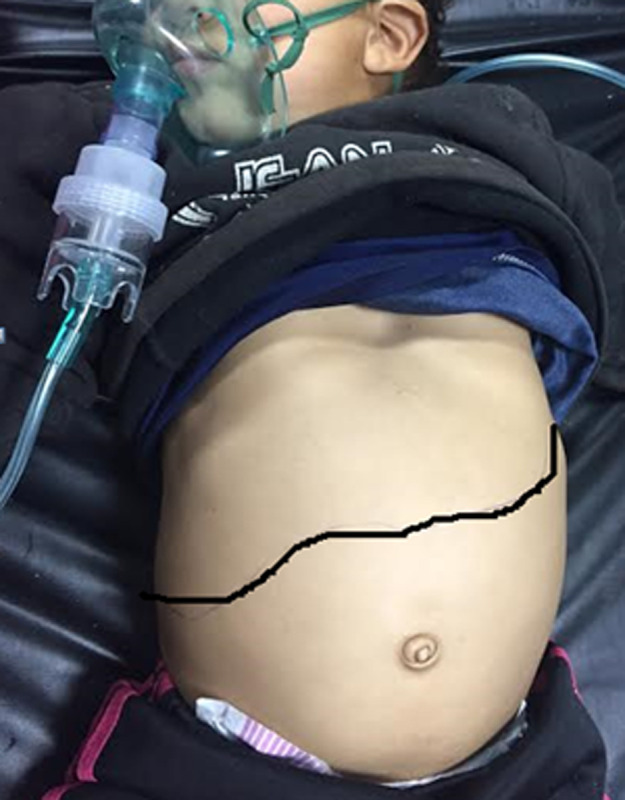
the patient presented an important hepatomegaly

**Figure 2:**
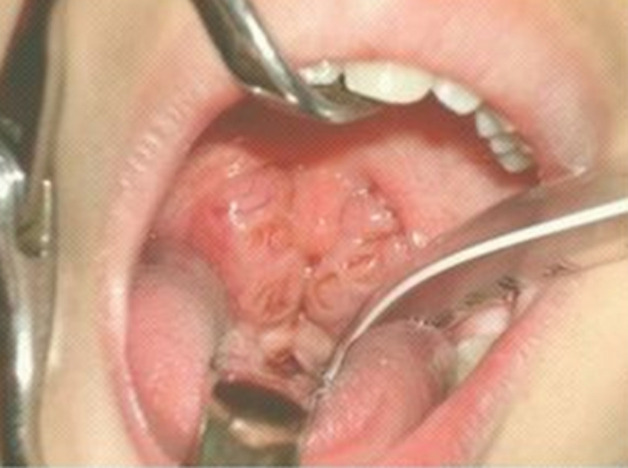
oropharyngeal examination revealing stage 4 adenotonsillar hypertrophy

**Clinical findings:** at admission, the child was conscious with severe signs of respiratory distress made of elevated breathing rate at 35 breaths per minute, oxygen saturation of 80% without oxygen therapy and 96% under oxygen therapy, nose flaring, and intercostal retraction. The body temperature was 38 degrees Celsius. The arterial tension was at 100/60 mmHg. Clinical examination revealed a spontaneous right jugular vein distension, snoring sounds on pulmonary auscultation, and important hepatomegaly (Figure 1). A head and neck examination revealed an important adenotonsillar hypertrophy (Figure 2).

**Timeline of the current episode:** symptoms evolved for 2 weeks.

**Figure 3:**
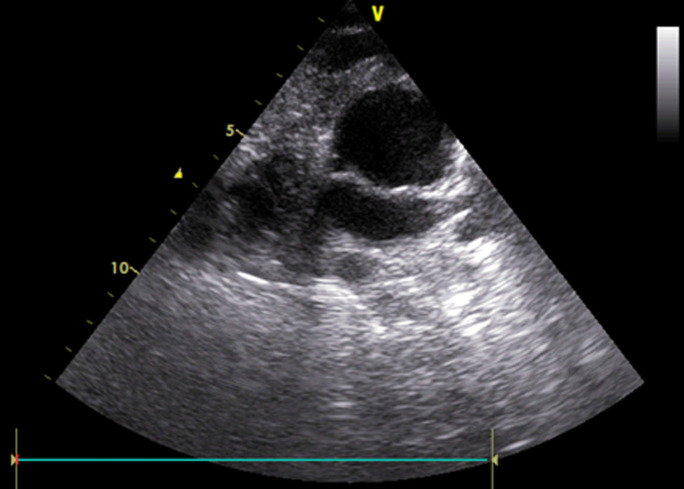
trans-thoracic echocardiogram revealed at admission an important distention of the right ventricle

**Diagnostic assessment:** chest radiograph revealed cardiac enlargement. The 12-lead electrocardiogram was suggestive of right heart cavities distension. The trans-thoracic echocardiogram showed enlargement of the right cavities (right ventricle and right atrium) (Figure 3). The right ventricular systolic strain was 12 mm. A tricuspid S´ wave was present at a speed of 0.7 cm/s and a tricuspid insufficiency was identified. The systemic pulmonary artery pressure had an estimated value of 64 mmHg. Both systolic and diastolic functions of the left ventricle were normal, with no distension or hypertrophy. No heart malformations have been found. Laboratory findings have revealed an elevated pro-brain natriuretic peptide (pro-BNP) level at 390 ng/l. C-reactive protein was at 17 mg/l. renal and hepatic functions were normal.

**Diagnosis:** a final diagnosis of right heart failure and pulmonary hypertension secondary to adenotonsillar hypertrophy has been established.

**Therapeutic interventions:** the management of the patient was based on oxygen therapy, administration of intra-venous diuretics (Furosemide 40 mg), and sildenafil 10 mg. An adenotonsillectomy has been performed.

**Figure 4:**
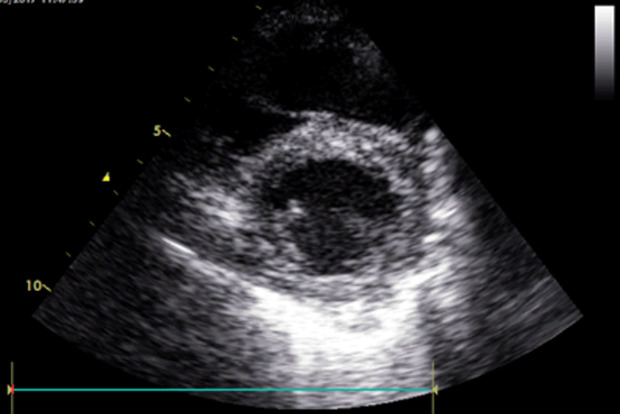
follow-up transthoracic echocardiogram after adenotonsillectomy, revealing diminished right ventricle dimensions

**Follow-up and outcome of interventions:** the follow-up has shown a complete resolution of initial symptoms one month after surgery. The follow-up echocardiogram revealed a normal systolic function of the right ventricle. This latter showed normal dimensions (Figure 4).

**Patient perspective:** the patient and his parents were satisfied.

**Informed consent:** informed written consent was obtained from the patient´s mother.

## Discussion

In this case report, we report a 3-year-old boy with right cardiac failure and pulmonary hypertension due to obstructive sleep apnoea syndrome (OSAS) secondary to bilateral tonsil hypertrophy. OSAS occurs in 3% of children [5]. Many revised diagnostic criteria for the diagnosis of OSAS exist [6]. In children, one of the following findings should exist to suggest a diagnosis of OSAS: labored/obstructed breathing, snoring, or daytime consequences [7]. In our case, labored breathing was evident at admission and was highly suggestive of OSAS.

Pulmonary hypertension (PH) and right heart failure are rarely reported complications of OSAS [7]. The occurrence of pulmonary hypertension is thought to be related to pulmonary artery constriction due to hypercapnia, chronic hypoxia, and acidosis all of which are present in cases of OSAS [7]. In the pediatric population, OSAS may have more severe complications such as systemic hypertension, growth failure, cor pulmonale, and even death [8]. PH classically shows a progressive evolution and is most often, as in our case, revealed by a rapid onset of cardiac decompensation. This phenomenon is explained by the unique physiology of the pulmonary vascular bed. In addition, the pulmonary vasculature is known for its very distensible nature, thus resulting in minimal changes in pulmonary artery pressure [9].

As in our case, many explorations are necessary to establish a correct diagnosis. Polysomnography is the gold-standard test for the diagnosis of OSAS. However, it is rarely applicable in the pediatric population, and the use of validated diagnostic questionnaires and clinical examination is more often the rule [8]. Echocardiography is a non-invasive test that enables the analysis of many cardiac and pulmonary parameters including diastolic and systolic heart functions in addition to the use of Doppler and Color Doppler techniques. Tissue Doppler Imaging (TDI) is an interesting, new technique enabling the detection of subclinical functional alterations of the heart [8]. The global ventricle function can be assessed using the myocardial performance index (MPI) [8].

On the therapeutic level, the pathophysiology of PH helps establish a basis of management, which is to reduce right ventricular afterload and thereby improve ventricular function and cardiac output. Pulmonary artery pressure per se should not be lowered. Medical therapies should be initiated to improve cardiac function before undergoing surgery for adenotonsillar hypertrophy or anesthesia [9]. The medical therapy of PH in these patients is based on pulmonary vasodilators such as prostacyclin, calcium channel blockers, and phosphodiesterase inhibitors [9]. According to the American Academy of Pediatrics, an adenotonsillectomy is indicated as a first-line management of OSAS secondary to adenotonsillar hypertrophy [3]. The European Respiratory Society is another society that indicates that adenotonsillectomy is indicated in cases of cor pulmonale pulmonary and hypertension [10]. As in our case, children with severe OSAS, in the presence of right heart failure and/or pulmonary hypertension are a frank indication for adenotonsillectomy [7].

## Conclusion

In children, adenotonsillar hypertrophy may be the cause of OSAS and thus be responsible for severe PH and right heart failure. Adenotonsillectomy has an important effect on cardiac function and pulmonary vasculature. However, surgery should be preceded by correct medical therapy. Our reported case should be a reminder for otolaryngologists, general pediatricians, cardiologists, and pediatric cardiologists to recognize this particular condition.
